# Obstructive Sleep Apnea With or Without Excessive Daytime Sleepiness: Clinical and Experimental Data-Driven Phenotyping

**DOI:** 10.3389/fneur.2018.00505

**Published:** 2018-06-27

**Authors:** Sergio Garbarino, Egeria Scoditti, Paola Lanteri, Luana Conte, Nicola Magnavita, Domenico M. Toraldo

**Affiliations:** ^1^Department of Neuroscience, Rehabilitation, Ophthalmology, Genetics and Maternal/Child Sciences, University of Genoa, Genoa, Italy; ^2^Department of Health Sciences, University of Genoa, Genoa, Italy; ^3^Institute of Clinical Physiology, National Research Council (CNR), Lecce, Italy; ^4^Department of Neurological Science, G. Gaslini Institute, Genoa, Italy; ^5^Interdisciplinary Laboratory of Applied Research in Medicine (DReAM), “V Fazzi” University Hospital, ASL Lecce, Lecce, Italy; ^6^Department of Biological and Environmental Sciences and Technologies, University of Salento, Lecce, Italy; ^7^Institute of Public Health, Università Cattolica del Sacro Cuore, Rome, Italy; ^8^Rehabilitation Department, Cardio-Respiratory Care Unit, “V Fazzi” Hospital, ASL Lecce, Lecce, Italy

**Keywords:** continuous positive airway pressure, excessive daytime sleepiness, hypoxia, obstructive sleep apnea, phenotype, sleep

## Abstract

**Introduction:** Obstructive sleep apnea (OSA) is a serious and prevalent medical condition with major consequences for health and safety. Excessive daytime sleepiness (EDS) is a common—but not universal—accompanying symptom. The purpose of this literature analysis is to understand whether the presence/absence of EDS is associated with different physiopathologic, prognostic, and therapeutic outcomes in OSA patients.

**Methods:** Articles in English published in PubMed, Medline, and EMBASE between January 2000 and June 2017, focusing on no-EDS OSA patients, were critically reviewed.

**Results:** A relevant percentage of OSA patients do not complain of EDS. EDS is a significant and independent predictor of incident cardiovascular disease (CVD) and is associated with all-cause mortality and an increased risk of metabolic syndrome and diabetes. Male gender, younger age, high body mass index, are predictors of EDS. The positive effects of nasal continuous positive airway pressure (CPAP) therapy on blood pressure, insulin resistance, fatal and non-fatal CVD, and endothelial dysfunction risk factors have been demonstrated in EDS-OSA patients, but results are inconsistent in no-EDS patients. The most sustainable cause of EDS is nocturnal hypoxemia and alterations of sleep architecture, including sleep fragmentation. These changes are less evident in no-EDS patients that seem less susceptible to the cortical effects of apneas.

**Conclusions:** There is no consensus if we should consider OSA as a single disease with different phenotypes with or without EDS, or if there are different diseases with different genetic/epigenetic determinants, pathogenic mechanisms, prognosis, and treatment.The small number of studies focused on this issue indicates the need for further research in this area. Clinicians must carefully assess the presence or absence of EDS and decide accordingly the treatment. This approach could improve combination therapy targeted to a patient's specific pathology to enhance both efficacy and long-term adherence to OSA treatment and significantly reduce the social, economic, and health negative impact of OSA.

## Introduction

Obstructive sleep apnea (OSA) is a sleep-related breathing disorder characterized by repetitive episodes of partial (hypopneas) or complete (apnea) obstruction of the pharyngeal airway, causing impaired gaseous exchange, varying degrees of hypoxia, and hypercapnia, which are usually terminated by brief arousals from sleep. The repetitive nature of apneas and hypopneas results in frequent arousals (sleep fragmentation) and disturbed sleep architecture, that are traditionally considered to contribute to the prominent—albeit not universal—symptom of chronic excessive daytime sleepiness (EDS) found in these patients (1). The American Academy of Sleep Medicine (AASM) defines EDS as the inability to maintain wakefulness and alertness during the major waking episodes of the day, with sleep occurring unintentional or at inappropriate times almost daily for at least 3 months (2). The Epworth Sleepiness Scale (ESS) is the most widely used clinical tool to evaluate subjective trait sleepiness based on a questionnaire testing individual dozing off attitudes (3). Despite its simple, inexpensive, and wide application, the ESS poorly correlates with OSA severity at individual level, and with objective tests of EDS, and is also open to reporting bias and confounding factors such as age, gender, psychological factors, and fatigue (4). Objective tools include in-laboratory—and more expensive and laborious—approaches (Maintenance of Wakefulness Test—MWT; Multiple Sleep Latency Test—MSLT) validated for specific diagnostic (MSLT to characterize suspected hypersomnias of central origin) or safety-related and medical-legal (MWT to address individual ability to resist sleep in monotonous conditions) purposes, and several non-validated psychomotor tests including simulated driving (5). Among signs/symptoms of AASM diagnostic criteria of OSA, in addition to EDS, is included fatigue (2): although frequently noted in combination in OSA and in comorbidity of OSA with other diseases, fatigue is distinct from EDS, and is a complex symptom related to the perception of lack of energy. Distinguishing sleepiness from fatigue can be difficult even for the most astute clinicians, but the use of assessment tools for EDS as well as for fatigue, including the Fatigue Severity Scale—FSS, and other rating scales, may assist in screening/diagnosis (6).

OSA is a prevalent condition affecting 3–7% of adult men and 2–5% of adult women in the general population (7), but these figures are expected to dramatically increase due to population aging and weight gain (8). Data indicate that OSA prevalence can be very high in selected populations, such as people affected by COPD (9) and even higher rates have been reported in some European Countries (49.7% men and 23.4% women with moderate-to-severe OSA in HypnoLaus Study cohort) (10). OSA is linked to an increased risk of related consequences including motor vehicle accidents, mood disruption, occupational injuries, and absenteeism (11–13), reduced quality of life (14), impaired cognition (15, 16), mental health problems (17). Clinical and epidemiological studies also found an independent association between OSA and all-cause mortality as well as cardiovascular disease (CVD) morbidity, and mortality, especially with regard to hypertension, arrhythmias, stroke, coronary artery disease, heart failure, and sudden death (18–21). Postulated mechanisms for CVD complications are complex and incompletely understood, but include OSA-associated chronic intermittent hypoxia (CIH), sleep fragmentation, chronic sympathetic activation, hypercoagulability, systemic inflammation, oxidative stress, and endothelial dysfunction (22–25). Although EDS has been regarded as a classical feature of OSA, population-based studies suggest that complaints of EDS are absent in many patients, especially where there is an association with CVD (26). This raises an important question on why sleepiness occurs in some patients but not others and whether no-EDS patients benefit from nasal continuous positive airway pressure (CPAP) therapy that has been demonstrated to be effective in OSA patients with EDS. Here, we will critically review the current knowledge on the issue of no-EDS OSA in terms of prevalence, response to therapy, and potential underlying determinants, trying to examine light and shadow of this often underestimated but clinically and epidemiologically relevant facet of OSA.

## Methods

Although there are few studies focused on no-EDS OSA patients, we tried to critically review the current knowledge in the literature to assess the state of art of this new argument. Due to small data in both randomized and non-randomized clinical trials that investigate non-sleepy OSA patients and due to the lack of any standard therapy, we could not conduct a systematic review approach according to PRISMA guidelines. However, we examined different database such as PubMed, Medline, and EMBASE. Search terms included randomized control trial, non-randomized clinical trial, OSA, sleep apnea, sleepiness, EDS, and non-sleepy patients; inflammation, OSA therapies. Additional studies were identified by contacting clinical experts.

### The heterogeneity of OSA: excessive daytime sleepiness does not tell the full story

Diagnosis, assessment of severity, and management of OSA is based on the apnea hypopnea index (AHI) that is the number of apneas and hypopneas per hour of sleep, as determined by standard overnight polysomnography. According to the AASM, OSA is defined with an AHI ≥ 5, and is classified as mild with AHI of 5–15, moderate with AHI of 16–30, and severe with AHI > 30 (27). However, OSA is recognized as a complex and heterogeneous disease in symptoms, etiological factors, comorbidities, and clinical outcomes (28). Although a consensus on detailed principles for classification remains to be established, different phenotypes, as defined by the clustering of clinical, pathophysiologic, cellular, and molecular characteristics, have been increasingly identified in OSA and may represent potential prognostic and therapeutic categories of patients that may enable the development of personalized medicine in OSA (29).

In this context, a number of clinical features, such as obesity, insulin resistance, and EDS are often present in OSA patients, but there are also many OSA patients who are not—or are less—symptomatic, without complaints of EDS (26, 30, 31). This heterogeneity could determine variable consequences on pathophysiologic mechanisms, long-term outcomes and response to therapy, and significantly challenges patient risk-stratification, and accurate management.

However, the definition of EDS and hence the classification of sleepy subjects in OSA remains a controversial issue. Some authors questioned the subjective assessment of EDS because OSA patients may complain of fatigue, tiredness, and lack of energy rather than sleepiness itself (32). The correlation between ESS and MSLT measures of sleepiness has long been debated, and has been reported to be weak-to-moderate (3). Of note, EDS is not a symptom exclusively related to sleep-disordered breathing, but also, for example, to depression, insomnia, and metabolic conditions (33). Residual sleepiness in effectively treated OSA patients may still lead to significant socioeconomic burden, including driving and job-related accidents (34), reduced quality of life (35), neurocognitive impairment (36, 37), and all-cause and cardiovascular morbidity and mortality (38–42). Recently, in a historical cohort study including 10,149 participants followed-up for 68 months and evaluating the association between OSA-related factors other than AHI and risk of CVD outcomes, EDS resulted as a significant and independent predictor of the occurrence of CVD events and all-cause mortality (42). EDS proved to be an independent predictor of road accidents and near-miss accidents risk in an Italian field study on truck drivers (11). Furthermore, in the presence of OSA, EDS is associated with an increased risk of hypertension (43, 44), and glucose deregulation (45, 46). Therefore, EDS identification and characterization is important for sleep clinicians not only for the suspicion/diagnosis of sleep-disordered breathing, but also for determining appropriate treatment in order to prevent its detrimental health consequences.

Although EDS affects around 12% of the general population (47), and several studies report higher ESS scores in OSA patients compared with controls (48), other epidemiological reports have questioned the association between EDS and OSA (49, 50). Using data from the Wisconsin Sleep Cohort study, Young et al. estimated a prevalence of 19% of EDS, assessed with three subjective questions on sleepiness, among 30–60 year-old adults with OSA (AHI ≥ 5) (50). In a large US community-based cohort, a minority (<50%) of middle-aged and older subjects with moderate-to-severe sleep disordered breathing (AHI ≥ 15) reported subjective sleepiness (51). Conversely, a study conducted in Europe found up to 60% of OSA middle-aged patients as having EDS (48), and a retrospective cross-sectional study in an Asian middle-aged population documented a relatively high prevalence (87.2%) of EDS, assessed with the MSLT, among OSA patients (52). The discrepancy in the prevalence data might be explained by the complex interplay of several factors, including the use of subjective or objective measures of EDS, differences in sample sizes, patient selection, OSA severity, ethnicity, accompanying comorbidities, and many other—often still unrecognized—factors impacting OSA presentation and implication. For example, aging is associated with a reduction in the symptom of EDS in male patients with OSA (53), and with different factors contributing to sleepiness compared with middle-aged OSA populations (54), thus suggesting that OSA in the elderly may be a distinct phenotype where the impact and related therapeutic implication of EDS seem to be attenuated. Recently, by using symptom-based cluster analysis in order to characterize the clinical presentations of OSA, the conventional figure of OSA symptomatic, sleepy patients was not the dominant phenotype in the cohorts analyzed, and patients without complaint of hypersomnolence (“minimally symptomatic, non-sleepy”) had a high prevalence of CV comorbidities (26, 55).

Therefore, the identification of less symptomatic and no-EDS OSA subjects may be particularly important in the clinical practice because of the high prevalence and the different association with comorbidities that may require tailored treatment.

### The OSA dilemma: how to treat No-EDS patients

Nasal CPAP therapy is the treatment of choice for severe OSA. CPAP keeps the upper airway open during sleep, and thus counteracts the negative suction pressure during inspiration that promotes upper airway collapse in OSA patients, and consequently normalizes sleep disturbance (56), improving daytime sleepiness, sympathetic tone, and quality of life in patients with OSA (57–59). In addition, CPAP therapy can reduce blood pressure (60, 61), insulin resistance (62), and endothelial dysfunction (63). Observational studies also showed that CPAP use is associated with reduced risk of fatal and non-fatal CVD events in patients with severe and moderate OSA (64–66). However, the positive effects of CPAP therapy are not consistent across the spectrum of OSA patients. Variable results have been documented regarding CPAP effect on blood pressure, with clinically significant reduction observed mostly in hypertensive patients and in severe OSA (67, 68). Due to the high incidence of hypertension and CVD complications in OSA and the potential CVD benefit associated with blood pressure reduction, one could speculate that CPAP would be useful in all subjects with OSA, irrespective of sleepiness symptoms. However, short-term CPAP treatment did not significantly reduce blood pressure and CVD risk in patients with severe OSA but without daytime sleepiness (69–73). The recent MOSAIC (Multicenter Obstructive Sleep Apnoea Interventional Cardiovascular) trial randomized 391 patients with no history of EDS or daytime symptoms of OSA to receive either CPAP or standard care for 6 months. The primary outcomes were changes in ESS score and the vascular risk, as measured by the Pocock calculated 5-year fatal cardiovascular risk score. CPAP treatment was associated with cost-effective reduction in daytime symptoms but did not reduce calculated cardiovascular risk (74). No treatment effect of CPAP was found on risk markers of cardiac dysfunction (75, 76), and markers of systemic inflammation interleukin (IL)-6, IL-10, C reactive protein (CRP), tumor necrosis factor (TNF)-α (77). The feasibility of long-term CPAP treatment in this group represents another important issue that has been addressed by few studies yielding conflicting results. Indeed, while EDS has been suggested as one of the most important determinants of adherence to CPAP because the EDS relief encourages patients to use the therapy, no-EDS patients with OSA might be less motivated to comply with CPAP because they do not experience subjective benefit from the treatment. Although the few studies focused on no-EDS OSA patients yielded conflicting results, a good adherence to long-term CPAP therapy has been recently reported in no-EDS, moderate-to-severe OSA patients, where OSA severity and the presence of hypertension were predictors of CPAP adherence (78).

Therefore, the management of no-EDS subjects, which are an important proportion of OSA patients and are more likely to present CVD, still constitutes a critical challenge mainly about whether or not CPAP should be recommended in this group of asymptomatic or minimally symptomatic subjects. Differently from OSA patients with EDS, the available evidence do not convincingly support CPAP use in no-EDS patients with OSA, and there is no clearly established rationale for treatment of such patients.

It should be acknowledged, however, that some clinical trials in no-EDS OSA patients found that long-term (>1 year) CPAP treatment is associated with a small reduction of blood pressure in hypertensive patients (79, 73), and of CVD events in patients with coronary artery disease after adjustment for baseline comorbidities (80). However, the magnitude of these effects was less than in the EDS subjects. Furthermore, in accordance with a *post-hoc* analysis in a primary prevention trial of the effects of CPAP on the incidence of hypertension and CVD events in no-EDS patients (69), CPAP effectiveness was evident only in patients who used CPAP for four or more hours per night, suggesting that a high level of CPAP use for a longer time is needed for CPAP to be effective in no-EDS patients. Although the magnitude of CPAP effect on blood pressure is modest, some authors advocate that even small reducing effects on blood pressure in the range of 2 mmHg may be clinically relevant as they have been associated with a reduction in the incidence of CVD (81). Besides primary endpoints, “intermediate” endpoints, which are considered as predictors of cardiovascular risk, might be improved by CPAP therapy. Six-month CPAP has been found to beneficially impact on endothelial dysfunction in asymptomatic patients, as assessed by flow-mediated dilatation, with longer nightly usage of CPAP associated with larger improvements (82). The improvement in endothelial function suggests that patients with OSA might have increased CVD risk and may benefit from CPAP treatment in terms of cardiovascular risk reduction. Accordingly, compared to well-matched control subjects without OSA, no-EDS OSA patients displayed elevated circulating levels of total, platelet-derived, and leukocyte-derived micro particles, an important link between OSA and pro-atherogenic mechanisms such as vascular inflammation, thrombosis, endothelial dysfunction, and atherosclerosis (83). Many OSA patients with CVD, such as chronic heart failure, do not exhibit EDS due to higher sympathetic activity leading to alertness (55, 84). A recent meta-analysis of randomized controlled trials found that the left ventricular ejection fraction, a marker of heart failure risk and status, increased significantly after CPAP treatment of OSA patients with heart failure (85). Therefore, caution should be used in deciding to treat or not patients solely based on EDS as well as about discarding the benefits of CPAP on the basis of the available limited evidence. It is also important to take into account the effects of potential confounding and/or etiological factors characterizing OSA, including cardiovascular and metabolic comorbidities that are known to influence the phenotypic expression of OSA and may mask or reduce the beneficial effects of CPAP treatment.

Large randomized studies with long-term follow-up are needed to assess the impact of CPAP on CVD outcomes in OSA patients without EDS, thus providing more solid foundation to guide therapeutic decisions. An ongoing randomized controlled trial will contribute to address this critical issue (86). An important step forward in the understanding of treatment response by EDS vs. no-EDS patients with OSA is represented by a more detailed identification of factors and potential pathophysiological mechanisms contributing to differentiate and identify these two groups of patients.

### Predictors of EDS: insight from human studies

Several studies attempted to investigate the factors that could differentiate between patients with EDS vs. those without EDS in order to identify correlates and possible predictors of these two phenotypic presentations. Many studies, which were performed in sleep-clinic or community-dwelling populations, compared a number of variables (demographics, polysomnography, comorbidities, metabolic factors, etc.) in patient groups with either clear-cut EDS or no-EDS, as assessed by ESS score and/or MSLT. The results are difficult to compare, owing to differences in epidemiologic design, degree of OSA, ethnicity, statistical methodology, and tools used to evaluate EDS among different studies. However, they provide an important insight into this phenomenon (Table 1).

**Table 1:** Differences between EDS and no-EDS patients with OSA as reported by literature studies.

**Author**	**Country**	**Study design**	**Number of subjects**	**Gender**	**OSA severity**	**Sleepiness assessment**	**Variables examined**	**Main differences between EDS and no-EDS**
Kapur (51)	USA	Cross-sectional	*n* = 1,115 (no-EDS *n* = 605, EDS *n* = 510)	Male (66%)	Moderate-severe (AHI ≥ 15)	ESS	Demographics, medical data and sleep complaints	EDS subjects were younger, more obese, and had more respiratory diseases, sedative use, sleep complaints, lower self-reported sleep hours
							Polysomnographic data	EDS subjects had higher AHI, hypoxemia, and lower oxygen saturation in REM and NREM sleep
Mediano (87)	Spain	Cross-sectional	*n* = 40 (no-EDS *n* = 17, EDS *n* = 23)	Male (100%)	Severe (AHI > 30)	ESS and MSLT	Polysomnographic data	EDS subjects had lower sleep latency, greater sleep efficiency, and lower nocturnal oxygenation. Nocturnal hypoxemia resulted a major determinant for sleepiness
Roure (88)	Spain	Cross-sectional	*n* = 2,882 (no-EDS *n* = 1,233, EDS *n* = 1,649)	Male (85%)	Severe (AHI > 30)	ESS	Demographic data	EDS subjects were younger and highly smoker
							Polysomnographic data	EDS subjects had lower sleep latency, greater sleep efficiency, longer total sleep time, less light sleep, increased SWS, slightly higher AHI, arousal index, and lower nocturnal oxygenation
Oksenberg (89)	Israel	Retrospective	*n* = 644 (no-EDS *n* = 242, EDS *n* = 327)	Male (84.5%)	Severe (AHI > 30)	ESS	Demographic and clinical data	EDS subjects were younger, more obese, and had lower percentage of hypertension
							Polysomnographic data	EDS subjects had a significant worsening of sleep-related respiratory variables (higher AI and AHI, lower minimum SaO_2_ in REM and NREM, higher snoring loudness), and sleep architecture variables (shorter sleep latency, lower percentages of SWS, higher number of short arousals, higher arousal index, higher number of awakenings). AI was found as a prognostic factor for EDS
Chen (90)	China	Cross-sectional	*n* = 1,035 of which 76% OSA	Male (84%)	Mild-to-severe	ESS	Polysomnographic and clinical data	Nocturnal hypoxemia resulted a major determinant of EDS, followed by body mass index and AHI
Montemurro (84)	Canada	Cross-sectional	*n* = 91 (no-EDS *n* = 60, EDS *n* = 31)	Male (78%)	Severe (AHI > 30)	ESS	Polysomnographic data	The no-EDS group had a higher AHI and arousal index and lower mean SaO_2_ than the EDS group. The no-EDS group had higher sympathetic activity as reflected by higher very low frequency heart rate variability during sleep
Bravo (91)	Spain	Cross-sectional	*n* = 50 (no-EDS *n* = 22, EDS *n* = 28)	Male (100%)	Moderate-severe (AHI ≥ 20)	ESS and MSLT	Plasma inflammatory markers	IL-6, TNF-α, ICAM-1 were not different between EDS and no-EDS subjects. Borderline lower levels of 8-iso-PGF2α in no-EDS vs. EDS
Koutsourelakis (92)	Greece	Cross-sectional	*n* = 915 of which 76.8% OSA (no-EDS *n* = 561, EDS *n* = 354)	Male (72.2%)	Moderate-severe (RDI > 5)	ESS	Demographic and clinical data	Depression and diabetes were important predictos of EDS, followed by COPD, stroke, heart disease, alcohol use, and BMI
							Polysomnographic data	OSA severity was the most powerful predictor of EDS
Uysal (93)	USA	Cross-sectional	*n* = 200	Male (99%)	Moderate-severe (AHI ≥ 15)	ESS	Polysomnographic data	Combined conventional hypoxemic measures predicted EDS in OSA patients only with AHI ≥ 50
Sun (94)	China	Cross-sectional	*n* = 80 (no-EDS *n* = 48, EDS *n* = 32)	Male (88%)	Mild-to-severe (AHI > 5)	ESS and MSLT	Demographic data	Compared to the no-EDS group, the EDS group had a significantly greater BMI
							Polysomnographic data	Arousal index, nocturnal hypoxemia, and REM sleep latency were independent predictors of EDS
Seneviratne (52)	Singapore	Cross-sectional	*n* = 195 (no-EDS *n* = 25, EDS *n* = 170)	Male (89%)	Mild-to-severe (RDI > 5)	MSLT	Demographic data	Compared to the no-EDS group, the EDS group had a younger age
							Polysomnographic data	The EDS group had more severe OSA. Arousal index, higher sleep efficiency, and severe snoring were independent predictors of EDS
Wang (95)	China	Retrospective	*n* = 338 (no-EDS *n* = 109, EDS *n* = 229)	Male (76%)	Moderate-severe (AHI ≥ 15)	ESS	Demographic and clinical data	Compared to the no-EDS group, the EDS group had younger age and higher diastolic blood pressure
							Polysomnographic data	The EDS group had more severe OSA and a higher nocturnal hypoxia
Barcelò (45)	Spain	Cross-sectional	*n* = 44 (no-EDS *n* = 22, EDS *n* = 22), matched for age, BMI, and AHI	Male (100%)	Severe (AHI > 30)	ESS and MSLT	Clinical data	EDS subjects showed anomalies in plasma levels of glucose, insulin, and HDL-cholesterol, and IR independent of BMI. 3-month CPAP therapy improved both EDS and IR in EDS subjects
							Polysomnographic data	Despite similar AHI, EDS subjects had higher nocturnal hypoxemia than no-EDS subjects
Nena (46)	Greece	Cross-sectional	*n* = 50 (no-EDS *n* = 25, EDS *n* = 25)	Male (86%)	Severe (AHI > 30)	ESS	Clinical data	EDS group was associated with hyperglycemia, hyperinsulinemia, and IR
Huang (96)	China	Cross-sectional	*n* = 175 (no-EDS *n* = 56, EDS *n* = 119)	Male (72%)	Severe (AHI > 30)	ESS	Clinical data	Compared to the no-EDS group, EDS group showed higher prevalence of metabolic syndrome
							Polysomnographic data	EDS group showed higher nocturnal hypoxia
Saaresranta (55)	European countries and Israel	Cross-sectional	*n* = 6,555 (no-EDS *n* = 3,644, EDS *n* = 2,911)	Male (75.4%)	Mild-to-severe (AHI > 5)	ESS	Demographic and clinical data	EDS groups were slightly younger and had lower prevalence of cardiovascular diseases
							Polysomnographic data	EDS groups had a more severe OSA

*AHI, apnea-hypopnea index; AI, apnea index; BMI, body mass index; COPD, chronic obstructive pulmonary disease; EDS, excessive daytime sleepiness; ESS, epworth sleepiness scale; ICAM-1, intercellular adhesion molecule-1; IL-6, interleukin-6; IR, insulin resistance; NREM, non-rapid eye movement; PGF, prostaglandin F; RDI, respiratory disturbance index; REM, rapid eye movement; SWS, slow-wave sleep; TNF-α, tumor necrosis factor-α*.

Male gender, younger age, higher body mass index have been frequently associated with the presence of EDS (33, 51, 87). Either sleep apnea or sleep fragmentation or both have been classically regarded as the predominant mechanisms leading to EDS in OSA (1, 97). According to earlier studies, one of the hypothesized mechanisms underlying EDS is the worse quality of nocturnal sleep due to the obstructive events, because several parameters indicative of alterations of sleep architecture including sleep fragmentation (e.g., sleep latency, sleep efficiency, slow wave, and REM sleep, arousal index, etc.) and/or sleep deprivation (e.g., total sleep time) were associated with EDS in OSA patients (49, 98, 99). Other authors did not find an independent association of sleep fragmentation with EDS (87, 88) and considered the presence of EDS as a result of nocturnal hypoxemia, as evidenced by several indices of oxygenation, mainly in severe OSA (87, 90, 93, 100, 101). In a small cohort of severe OSA patients, EDS subjects displayed shorter sleep latency, higher sleep efficiency, and worse nocturnal oxygenation than age-, BMI-, and gender-matched subjects without EDS. Here, no significant difference in AHI, arousal index, and sleep stage distribution could be found (87). The authors postulated that the peculiar pattern of sleep efficiency and latency in EDS patients is a consequence and not a cause of EDS and, in accordance with earlier reports (100), they conclude that hypoxemia may underlie EDS. These findings were confirmed and expanded in a larger cohort of 2,882 patients with severe OSA (AHI ≥ 30) with or without EDS (assessed using a combination of both ESS score and MSLT), demonstrating that EDS subjects sleep longer and more efficiently than patients without EDS, and are also characterized by only slightly increase in respiratory disturbances, as evidenced by higher AHI and lower nocturnal oxygenation, and by a marginal, albeit significant, degree of sleep fragmentation, as evidenced by an increase in arousal index. Therefore, in this study sleep apnea and fragmentation were not found as major clinically important determinants of EDS in OSA (88). However, by analyzing individual and combined hypoxemia variables in OSA cohorts with a wide range of OSA severity, Uysal et al. (93) found that EDS and hypoxemia were significantly associated only in the more severe patients (AHI > 50), whereas non-hypoxemic factors might be responsible for EDS in patients with milder degree of OSA. Using the joint ESS and MSLT criteria proposed by Mediano et al. (87) to identify EDS and no-EDS patients, and simultaneously assessing a large number of nocturnal determinants of EDS, in a small cohort including patients with mild sleep apnea Sun et al. observed a more severe sleep AHI and hypoxemia, sleep fragmentation (characterized by increased arousals), low quality of sleep, and increased pressure of nocturnal sleep drive (as reflected by increased total sleep and slow wave sleep) in EDS compared with no-EDS patients (94). Similarly, using the MSLT, Seneviratne et al. (52) showed that severe snoring, higher sleep efficiency, and an increase in total arousals seemed to be the most useful predictors for EDS in patients with OSA.

By assessing differences in demographic and polysomnographic variables between EDS and no-EDS severe OSA patients, Oksenberg et al. (89) found that, compared with no-EDS patients, EDS patients had a more severe OSA, as documented by worse sleep-related breathing parameters such as apnea index (AI), AHI, minimal SaO_2_ in rapid eye movement and not rapid eye movement sleep, and by disturbed sleep patterns characterized by lighter and more fragmented sleep. At the multivariate analysis, AI was found as a significant negative prognostic factor for EDS.

#### Role of comorbidities

Although the degree of sleep fragmentation and hypoxemia are the two most commonly accepted explanations for EDS in OSA, the literature reports a wider variety of factors in addition to sleep disturbances that can drive sleepiness and can explain why many OSA patients do not report EDS (33, 51, 92, 102). Indeed, it has become increasingly clear that the pathogenesis of EDS in OSA is multifactorial, and factors other than sleep apnea and fragmentation may contribute to explain EDS in OSA. Supportively, EDS can persist after significant reduction in sleep-disordered breathing with effective CPAP (103), and, as stated above, studies in clinic and general population samples suggested that the relationship between OSA severity and subjective sleepiness is weak or even absent (32, 51).

Besides polysomnographic measures, in a cross-sectional study in a subsample of the Sleep Heart Health Study self-reported common comorbid conditions in OSA including insomnia, partial sleep deprivation, periodic limb movements, and obstructive pulmonary diseases, especially COPD, were found to be risk factors for EDS (51). In a sleep clinic-based sample, the most powerful predictors of EDS are OSA severity, depression and diabetes, followed by the presence of comorbidities such as COPD, a history of heart disease and stroke, body mass index, and alcohol use (92). Although these comorbid conditions are independently correlated with EDS, their role in the development and treatment of sleepiness in OSA needs to be further addressed.

Since EDS persisted in many patients despite significant reduction in sleep-disordered breathing with CPAP therapy, some authors evaluated potential predictors of this residual sleepiness in CPAP-treated OSA patients (102). In keeping with previous results (92), a history of depression, diabetes, heart disease, and an initial lower OSA severity and increased ESS score significantly and independently predicted the occurrence of residual sleepiness in CPAP-treated patients (102), suggesting the importance of considering these clinical factors in anticipating the response to therapy in terms of resolution of EDS.

#### Role of metabolic diseases

Interestingly, some of the clinical factors consistently found to be independently associated with EDS are impaired glucose metabolism and insulin resistance (45). Evidence suggest that EDS is an independent risk factor for diabetes in the general population (104) and in severe OSA (105). EDS patients with severe OSA exhibited worse nocturnal hypoxemia (mean and minimum oxygen saturation), and altered plasma levels of glucose, insulin, high density lipoprotein (HDL), as well as evidence of insulin resistance (higher HOMA index), compared with age-, OSA severity-, and body mass index-matched no-EDS patients which are instead similar to the healthy control group. Three-month CPAP improved both EDS and insulin resistance in EDS patients while no reduction was observed in no-EDS patients. Therefore, the presence of EDS seems to predict a favorable response to CPAP treatment in improving insulin resistance independently of obesity, thus representing a particular OSA phenotype (45). Accordingly, while levels of hCRP, a marker of systemic inflammation, and lipidemic profile did not differ between EDS and no-EDS OSA patients, EDS patients are characterized by hyperglycemia and insulin resistance compared to no-EDS ones (46). These results collectively suggest EDS as a surrogate marker useful to identify patients with OSA at increased risk of developing metabolic syndrome and diabetes. In agreement with these data, occurrence of metabolic syndrome was significantly higher in severe OSA patients (AHI ≥ 30/h) with EDS compared with no-EDS patients, and ESS score was an independent predictor of metabolic syndrome after adjustment for body mass index, currently smoking, alcohol consumption, AHI, and oxygen saturation (96). Postulated mechanisms underlying the association of EDS with impaired glucose homeostasis and insulin resistance involve the contribution of sleep fragmentation-associated increased sympathetic tone and adrenocortical activity (106) as well as of intermittent hypoxia (107), ultimately resulting in decreased insulin-mediated glucose uptake, and increased muscle glycogen breakdown, hepatic glucose output, and release of free fatty acids. In addition, intermittent hypoxia might induce inflammation, oxidative stress, and endothelial dysfunction, all contributing to the development of cardiometabolic diseases (108).

#### Role of genetic factors

Of note, many other potentially important factors not explicitly taken into account in these studies may explain the difference between EDS and no-EDS patients and even might have been confounding factors in previous studies. These factors include, but are not limited to, nutritional status, physical activity, body fat distribution, and genetic factors. Therefore, confirmation in large populations in whom these factors are adequately controlled is required. In this regard, complex interaction of subject genetic make-up with environmental factors may ultimately determine the susceptibility or not to develop sleepiness. Inter-individual phenotypic variation has been described in the arousal response to each apnea, and it has been hypothesized that no-EDS patients are less susceptible to the cortical effects of apneas, thus experiencing less sleep disruption (109, 110). Approximately 30–40% of the variance in the AHI has been estimated to be explained by genetic factors, and candidate gene and genome-wide association studies have highlighted that genetic factors may play a role not only conferring disease risk but also modulating the way individuals deal with the disease consequences, influencing pathways involved in AI index, propensity for hypoxemia and respiratory arousability, sleep–wake characteristics, craniofacial development, inflammation, ventilatory control (111, 112). Genetic variants found to be associated with OSA traits include polymorphisms in inflammatory cytokines, such as CRP, TNF-α, IL-6, glial cell line–derived neurotrophic factor (GDNF), involved in the ventilator control pathway, receptor of hypocretin/orexyn, a neurotransmitter with effects on sleep/wake regulation (112). Polymorphisms in the Apolipoprotein E gene have been shown to be associated with sleep parameters in OSA patients, emerging as potential modulators of the deleterious effects of intermittent hypoxia and sleep fragmentation on the sleep architecture (113): in particular, ε2 allele carriers showed longer sleep latency, lower sleep efficiency, higher arousal index, higher percentage of stage 1 and a lower percentage of stages 3+4, and spent more time awake, even after correction for potential confounders such as age, sex, and African ancestry and correlated lipid levels.

Another emerging mechanism that may predispose to EDS is represented by epigenetic changes (DNA methylation, post-translational modifications of histone proteins, chromatin remodeling, and transcriptional regulation by non-coding RNAs-miRNAs), i.e., changes in gene expression without a corresponding change in the DNA sequences that are highly modified in response to environmental factors. A recent large-scale DNA methylation analysis in peripheral blood mononuclear cells identified differentially methylated loci that were shown to correlate with both AHI and the patients' susceptibility to EDS, suggesting different epigenetic-dependent phenotypes. Among these epigenetic changes, natriuretic peptide receptor 2 (NPR2) hypomethylation, and speckled protein 140 (SP140) hypermethylation were associated with EDS in patients with OSA (114). Inferences on the causal role of epigenetic modifications in OSA have been provided by studies in rats where neonatal intermittent hypoxia induced DNA hypermethylation of antioxidant enzyme-encoding genes, such as that encoding superoxide dismutase 2, in the carotid body and adrenal medulla, resulting in oxidative stress. The authors suggested that these epigenetic modifications exaggerated hypoxic sensing and induce autonomic dysfunction in adult rats, including increased hypoxic ventilatory response and sleep apnea events (115, 116). Further investigations are required to validate and expand these results, and to clarify the cause-effect relationship between epi-/genetic factors and OSA, as well as the contribution to different OSA outcomes including EDS and even treatment responses.

### Inflammation and oxidative stress in the pathogenesis of EDS: animal models and therapeutic implication

An unresolved and intensively studied issue in OSA field regards the pathophysiological mechanism(s) linking sleep apnea with adverse consequences, mainly CVD as well as neurobehavioral complaints including EDS. The pathophysiologic mechanisms proposed for OSA include sympathetic hyperactivation, impairment of vasomotor reactivity, inflammation, oxidative stress, endothelial dysfunction, and metabolic disorders. Numerous comorbidities—including diabetes, CVD, and obesity—are associated with the disease, making difficult in clinical studies to determine whether comorbidities increase the propensity for adverse effects or whether OSA alone causes such effects. Therefore, animal models are instrumental in elucidating the pathophysiological mechanisms determining the consequences of OSA and to mimic some manifestations of OSA in humans under well-controlled experimental conditions and independently of comorbidities. Animal models used to mimic and study sleep-disordered breathing can be either spontaneous or induced (117). Spontaneous models for OSA have been documented in the English bulldogs and in pig, which were reported to have an abnormal narrowing upper airway anatomy. Those models may reproduce all the clinical features of human OSA, but their relatively low availability and mild hypoxemia have spurred interest in induced models. Because numerous clinical studies (described above) have suggested that hypoxemia may predict EDS and other consequences of OSA, researchers have developed animal models of frequent brief hypoxemic episodes to model the oxygenation patterns of moderate–severe OSA and examine the effects of sleep apnea oxygenation patterns on physiological processes associated with OSA. Invasive models reproduce OSA in tracheostomized animals installed with an intermittently blocked endotracheal tube (118).

Non-invasive models, such as the CIH model, easily reproduce the chronic repetitive hypoxia-reoxygenation process and have been widely used to evaluate various consequences of OSA. This model allows the evaluation of oxygen desaturation, hypercapnia, sustained hypoxia, and sleep fragmentation. Animals either breathe with a mask or are put in specific chambers or cages, where they intermittently breathe nitrogen-enriched air to produce hypoxia, alternating with oxygen or air for the reoxygenation (119). An important limitation of this model is the lack of an upper airway occlusion that does not allow to evaluate the potential consequences of breathing efforts.

The CIH models have shown to recapitulate protracted hypersomnolence, elevation in blood pressure, central nervous system damage, and anomalies in glucose and lipid metabolism (120). In particular, selective neuronal cell losses occur in brain regions mediating sleep–wake regulation (120, 121), accompanied by activation of several pro-inflammatory pathways, such as platelet-activating factor, release of glutamate, induction of cyclooxygenase (COX)-2, release of proinflammatory cytokines, as well as by oxidative modifications, such as nitration, lipid peroxidation, and carbonylation in many brain regions, including wake-active regions and the hippocampus (122–125). Several subcellular compartments seem to be involved in the production of reactive oxygen species (ROS), such as mitochondria, endoplasmic reticulum, cellular membrane, lysosomes, peroxisomes, through enzymatic systems which include NADPH oxidase, xanthine oxidase, phospholipase A2, lipoxygenases, COX-2, and inducible nitric oxide synthases (iNOS) (126). Repetitive episodes of ischemia/reoxygenation in OSA patients could lead to dysfunction of mitochondria and endoplasmic reticulum and activation of NADPH oxidase, which may cause overproduction of ROS and following protein, lipid, and DNA peroxidation damage, resulting in substantial inflammatory response. In the CIH model of sleep apnea, iNOS has been found to contribute to CIH-driven nitration and lipid peroxidation injuries and to the inflammatory injury to wake-promoting regions of the brain, such as the laterobasal forebrain and posterolateral hypothalamus (124). In addition, genetic ablation or pharmacologic inhibition of NADPH oxidase, one of the major enzymes involved in the production of oxidants, prevented long-term hypoxia reoxygenation-induced hypersomnolence, the associated proinflammatory gene response (TNF-α, COX-2, iNOS), carbonylation, and lipid peroxidation injury to wake-active regions (125). Being also critically implicated in hypertension and ischemic heart disease, that are known adverse consequences of OSA, NADPH oxidase may represent an interesting pharmacotherapeutic target for both neurobehavioral and cardiovascular morbidities of OSA. Intriguingly, sex differences have been reported in the susceptibility to EDS in human subjects and, accordingly, animal models indicated less severe brain oxidative injuries and hypersomnolence in females in response to IH, probably due a neuroprotective role by estrogen (127).

Hypoxia can increase iNOS, NADPH oxidase, and other inflammatory and pro-oxidative mediators through the activation of the hypoxia-sensitive transcription factors hypoxia inducible factor (HIF)-alpha, nuclear factor-like 2 (Nrf2), activator protein 1 (AP1), and/or nuclear factor (NF)-κB, and triggering the mitogen-activated protein kinase (MAPK) signaling cascade (128, 129). In this regard, Ryan et al. previously reported that while sustained hypoxia leads to the activation of HIF-1, resulting in adaptative and protective responses, in cell culture models of IH as well as in OSA patients a preferential activation of inflammatory pathways regulated by NF-κB has been clearly demonstrated (130). However, the contributory role of each transcription factor may be tissue-specific (131).

Collectively, these results gathered from animal models of hypoxia are supportive of some potential mechanisms, including inflammation and oxidative stress, able to contribute to the pathophysiology of OSA and, in some case, EDS, suggesting also possible therapeutic targets to improve OSA consequences and residual EDS in persons treated for sleep apnea.

These experimental findings support the demonstrated efficacy of antioxidant therapy against neuronal injury (123), and the observation that OSA in humans is accompanied by activation of pro-oxidant enzymes, increased generation of free radicals and resultant oxidative stress (132). Correspondingly, a number of studies have also suggested that treatment with CPAP could attenuate oxidative stress levels in OSA patients (133, 134). However, other studies did not find a link between OSA and oxidative stress and questioned the positive results because of confounding factors, such as age, obesity, smoking, dietary habits, hypertension, diabetes, hyperlipemia, coronary heart disease, metabolic syndrome, and other concurrent comorbidities, which might *per se* augment oxidative stress (135).

**Figure 1:**
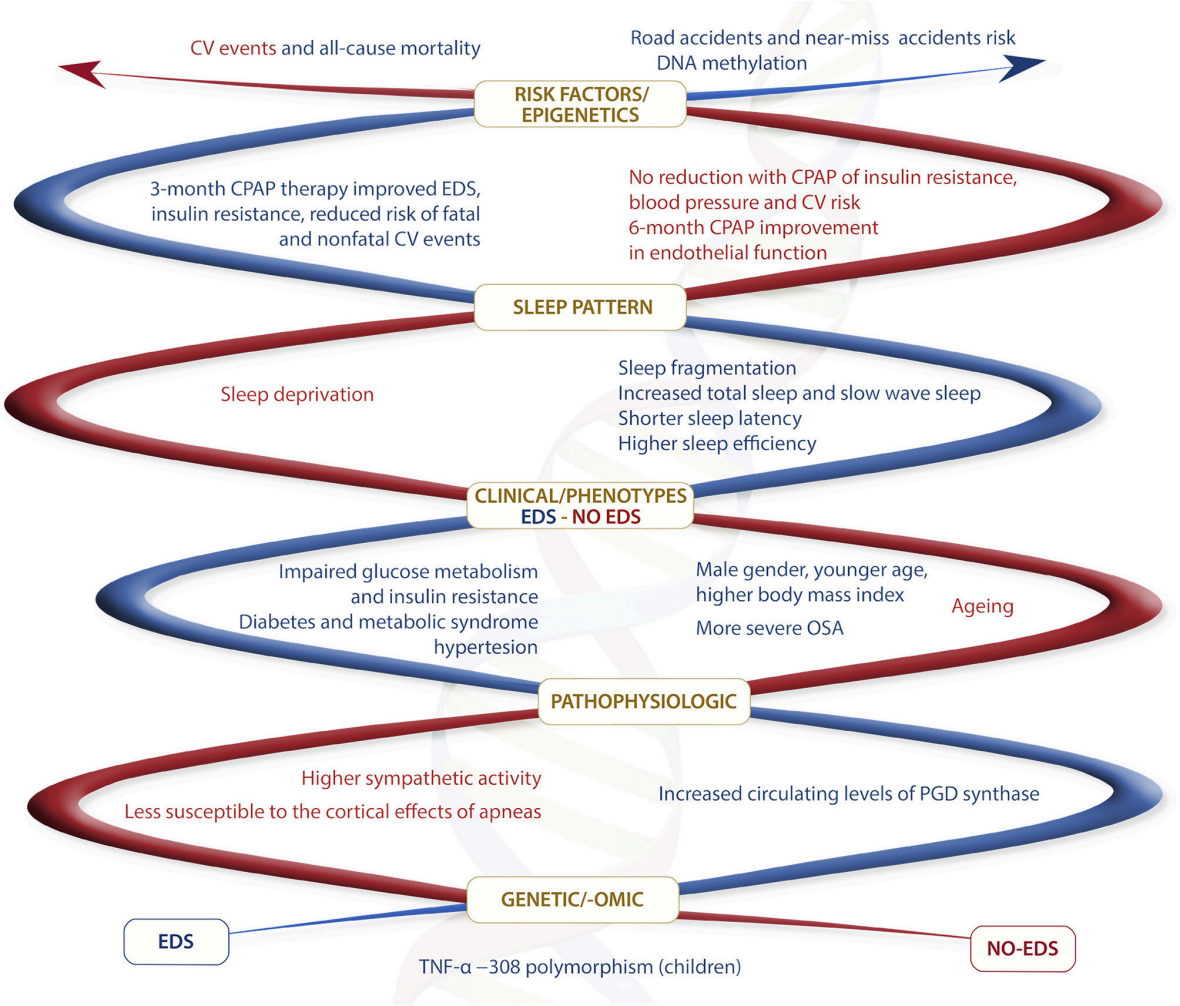
Summary of main different features between EDS and no-EDS OSA phenotypes. In red different features for no-EDS phenotype and in blue different features for EDS phenotype.

Several studies have also documented increased circulating levels of inflammatory and somnogenic cytokines including TNF-α, IL-6 as well as ICAM-1, in patients with OSA compared with controls, and a significant fall with effective CPAP treatment (91, 136). TNF-α gene polymorphisms (−308 G) seems to be associated with the magnitude of sleep latency and EDS in children OSA population (137, 138). Data on the association between levels of inflammatory markers and EDS are conflicting, with some studies reporting a significant correlation of EDS with the levels of inflammatory markers independently of comorbidities (139–141), and other studies that failed to document such an association (142). As stated in the case of oxidative stress, it should be taken into consideration that obesity, diabetes, and CV diseases are associated with inflammatory responses both at tissue and systemic levels, and many studies did not exclude these conditions that could have a major impact on the research outcomes. In particular, obesity-associated daytime sleepiness has been suggested to be mainly associated with metabolic factors and less with sleep apnea and sleep disruption, and to be mediated by inflammatory cytokines (143). Interestingly, Vgontzas et al. have shown a significant reduction in EDS with the TNF-α receptor antagonist etanercept in a pilot study, thus proposing TNF-α and IL-6 as mediators of EDS (144). It is known that COX-2 upregulation in the brain, as well as in other tissues, results in prostaglandin (PG) H2 synthesis that can be converted in wake-sleep and proinflammatory mediators PGE2 and PGD2, this last a somnogenic factor (145). In agreement with the observed intermittent hypoxia-induced COX-2 expression and activity, a pilot study has shown that circulating levels of PGD synthase are increased in OSA patients with EDS compared with no-EDS patients, thus suggesting a possible pathophysiological role for COX-2/PGD synthase/PGD2 signaling in EDS (146) (Figure 1).

These several mechanisms described above may lead to neurodegenerative diseases such as Alzheimer's disease (AD), Parkinson's disease (PD), dementia with Lewy bodies, multiple system atrophy (MSA), hereditary ataxias, and amyotrophic lateral sclerosis (ALS) (147). Indeed, some sleep disturbances including sleep-disordered breathing can be the first manifestation of the disease, during its early stages, and an independent risk factor for their development (5, 148).

Cell loss of the brainstem nuclei that modulates respiration, and dysfunction of pharyngeal, laryngeal, and diaphragmatic muscles can increase the risk for sleep-disordered breathing in neurodegenerative diseases (147). OSA may lead to neurodegenerative process with mild cognitive impairment and dementia (149) through chronic nocturnal hypoxemia (150, 151), abnormal cerebral hemodynamic (152), sleep fragmentation (153), mediation through CVD risk factors (e.g., hypertension, diabetes, inflammation), stroke (both clinical and subclinical) (154, 155), Aβ plaque build-up (156) and interaction with the APOE ε4 risk allele (157). Chronic intermittent hypoxia drives a number of the pathological mechanisms, including neuroinflammation, microangiopathy, and mitochondrial dysfunction related to enhanced Aβ deposition and tau phosphorylation. Aβ activates microglia and astrocytes and enhances oxidative imbalance, mediated by neuroinflammation with disruption of synaptic function and upregulating neuronal dysfunction and memory decline (158). A recent study reported elevated concentrations of phosphorylated-tau and Aβ 40, Aβ 42, and total Aβ levels in middle-aged cognitively normal OSA patients compared to healthy controls (159). Relatively young patients with moderate-severe OSA have elevated concentrations of tau and IL-6 in peripheral blood samples, compared to patients with mild OSA and healthy controls (160). Peripheral tau concentrations are correlated with AHI, suggesting that this biomarker may be associated with OSA severity. Higher cerebrospinal fluid total tau levels are associated with OSA and cognitive impairments (161).

A significant association has been found in PD between OSA and the disabling non-motor symptoms of cognitive dysfunction, as well as EDS. Both of these associations persisted in multivariate analyses after adjustment for several potential confounding factors. These relationships were independent of other sleep disorders frequently present in PD and associated with cognitive dysfunction (162).

Although not exhaustively, these mechanistic data may provide an interesting framework for a deeper understanding of the pathophysiology and different clinical manifestations of OSA, including sleepiness, and potential avenue(s) for developing preventive strategies and therapeutic interventions and for attenuating neurodegenerative processes, memory, and cognitive dysfunction among OSA patients, as well.

## Conclusions

OSA is a serious condition with major consequences and a troubling prevalence. CPAP therapy is the treatment of choice for severe OSA but its positive effects are not consistent across the spectrum of OSA patients. Currently, there is no method to predict which treatments will have the best outcomes in individual patients. Methods have been developed to quantify specific traits contributing to OSA. We tried to collect all data available in the literature about a specific group of OSA patients without one of the primary symptoms, EDS, to understand if this recently well-known clinical phenotype is only a clinical typing or is a different endotype or pathology.

Data are heterogeneous in sampling, different cohorts, no standardized diagnostic methods for EDS. A crucial issue remains the limitations of the tests used to evaluate EDS in OSA, with low statistical correlation and differences in terms of underlying mechanisms and prognostic value between objective and subjective measures of EDS. Therefore, studies are mandatory to more precisely address this debate and improve methods to distinguish EDS and no-EDS in OSA with clear consequences for the clinical diagnosis, management, and treatment of OSA. Furthermore, most studies of OSA pathophysiology do not consider specifically EDS vs. no-EDS or do consider but only in a small number of cases or not as a primary endpoint. Many studies of OSA genetic and genomics during the last 10 years do not take into account the absence of EDS in many OSA patients, but include other variants such as AHI, obesity, craniofacial malformation, smoking. However, to confirm and better understand the real differences between EDS and no-EDS phenotypes, future genomic, and genetic evaluations as well as the role of environmental factors are mandatory. EDS is so widespread in OSA patients that seems to look as a unique entity together with the definition of diagnosis but its real prevalence is very heterogeneous in different study sample. Nevertheless, the results obtained so far are tremendously encouraging for showing significant differences in many aspects of OSA clinical profile if EDS is present or not. These differences may be considered by clinicians and researchers for translation into a testing for OSA screening, diagnosis, treatment, and prognosis in EDS or no-EDS phenotypes.

In our opinion, there is ground to open new perspectives for the implementation of routine clinical care with different clinical approaches of OSA patients without EDS for OSA management (diagnosis, identification of biomarkers and genetic risk assessment, predisposition to disease development and progression, and response to treatment). This approach could improve to customize combination therapy targeted to a patient's specific pathology to enhance both efficacy and long-term adherence to OSA treatment and significantly reduce the social, economic, and health negative impact of OSA.

## Author contributions

SG and DT: study conception; ES, SG, PL, and DT: drafting manuscript; ES, SG, DT, PL, LC, and NM: revising manuscript content. All authors: approving final version of manuscript.

### Conflict of interest statement

The authors declare that the research was conducted in the absence of any commercial or financial relationships that could be construed as a potential conflict of interest.
